# Microscopic tumor foci in axillary lymph nodes may reveal the recurrence dynamics of breast cancer

**DOI:** 10.1186/s40880-019-0381-9

**Published:** 2019-06-19

**Authors:** Romano Demicheli, Marco Fornili, Patrizia Querzoli, Massimo Pedriali, Saverio Alberti, Christine Desmedt, Elia Biganzoli

**Affiliations:** 1 Unit of Medical Statistics, Biometry and Bioinformatics, Campus Cascina Rosa, Fondazione IRCCS Istituto Nazionale Tumori, Laboratory of Medical Statistics and Epidemiology, “Giulio A. Maccacaro”, Department of Clinical Sciences and Community Health, University of Milan, Via Vanzetti 5, 20133 Milan, Italy; 2 Laboratory of Medical Statistics and Epidemiology, “Giulio A. Maccacaro”, Department of Clinical Sciences and Community Health, University of Milan, Via Vanzetti 5, 20133 Milan, Italy; 3 Section of Surgical Pathology, Department of Morphology, Surgical and Experimental Medicine, University of Ferrara, 44121 Ferrara, Italy; 4 Operative Unit of Surgical Pathology, Azienda Ospedaliera – Universitaria, University of Ferrara, 44121 Ferrara, Italy; 5 Section of Medical Genetics, Department of Biomedical and Odontoiatric Sciences, Morphological and Functional Imaging, University of Messina, 98125 Messina, Italy; 6 Laboratory for Translational Breast Cancer Research, Department of Oncology, Katholieke Universiteit Leuven, Herestraat 49, 3000 Louvain, Belgium

Dear Editor,

In early breast cancer, the prognostic value of the number of macroscopically metastasized axillary nodes has been recognized in earlier reports [1]. However, the impact of microscopic tumour cell deposits on the survival outcome of early breast cancer patients is still debated [2]. This issue has gained increasing attention since the implementation of sentinel node biopsy in axillary node staging for tailoring breast cancer treatments, and the status of the single resected node would determine the clinical decision of whether or not to perform the axillary lymph node dissection [3]. Moreover, this issue raises more questions on whether to administer primary or adjuvant systemic treatment for patients with lymph nodes bearing isolated tumour cells (pN0[i+]) or micrometastases (pN1mi). Despite the wide debate on the clinical treatment dilemma encountered by early breast cancer patients with microscopic tumour cell deposits, the biology underlying different pathological presentations at microscopic level (pN0, pN0[i+], pN1mi) and the disease outcomes remain poorly known. In an attempt to shed some light on this topic, we have analyzed, in the context of dormancy-based metastasis development model [4], early breast cancer patients conventionally classified as pN0 (tumour foci with largest diameter ≤ 2 mm) [5] by systematically reassessing their tumor recurrence dynamics following primary tumour resection at a single institution.

The analyzed database [5] comprised of 377 early breast cancer patients who underwent mastectomy (67%) or conservative surgery (33%) and staged as pN0, based on the 6th edition of the Union for International Cancer Control/American Joint Committee on Cancer (UICC/AJCC) TNM classification [6]. Adjuvant systemic treatment was administered to 29% (chemotherapy, 8% and hormone therapy, 21%) of the patients, whereas 55% received surgery only and 16% had incomplete records. This study cohort had a median follow-up period of up to 8 years (range 1–13 years).

A total of 6676 axillary lymph nodes were surgically harvested, step-sectioned at every 200 μm (about 250 sections per patient), stained with haematoxylin and eosin (Fig. 1a, c), and reassessed by immunohistochemical analysis for cytokeratin expression (AE1-AE3-PCK26 antibody, Ventana, Tucson, AZ) (Fig. 1b, d). Immunohistochemistry was done with an automated immunostainer (Ventana NEXES Medical System, Tucson, AZ) run with Ventana kits (Strasbourg, France). Lymph node sections were independently examined by two pathologists, with the help of a computerized image analyzer morphometric system (EUREKA, Menarini Diagnostic, Florence, Italy). Accordingly, the pN0 patients were re-classified as: (1) pN0(i−), no detectable tumour deposits; (2) pN0(i+) (nanometastases), isolated tumour cells or foci with largest diameter ≤ 0.2 mm, (Fig. 1a, b); and (3) pN1mi (micrometastases), tumour foci with the largest diameter being between 0.2 and 2 mm (Fig. 1c, d). Clinical events such as tumor recurrence (local or distant), the occurrence of second primary (including contralateral breast primary), and death, occurring within the median follow-up time, were examined (Table 1). The recurrence dynamics was investigated by estimating the cause-specific hazard rates, i.e., the rates of recurrence of a specific event at a certain follow-up time. To estimate the hazard rate at different follow-up times, a discretization of the time axis in 6-month units was applied (therefore hazard rates were estimated in recurrences/6 months) and to obtain a smoothed pattern of the hazard rate values a kernel estimator was adopted [7]. This smoothed curve is graphically presented (Fig. 1e).

**Fig. 1:**
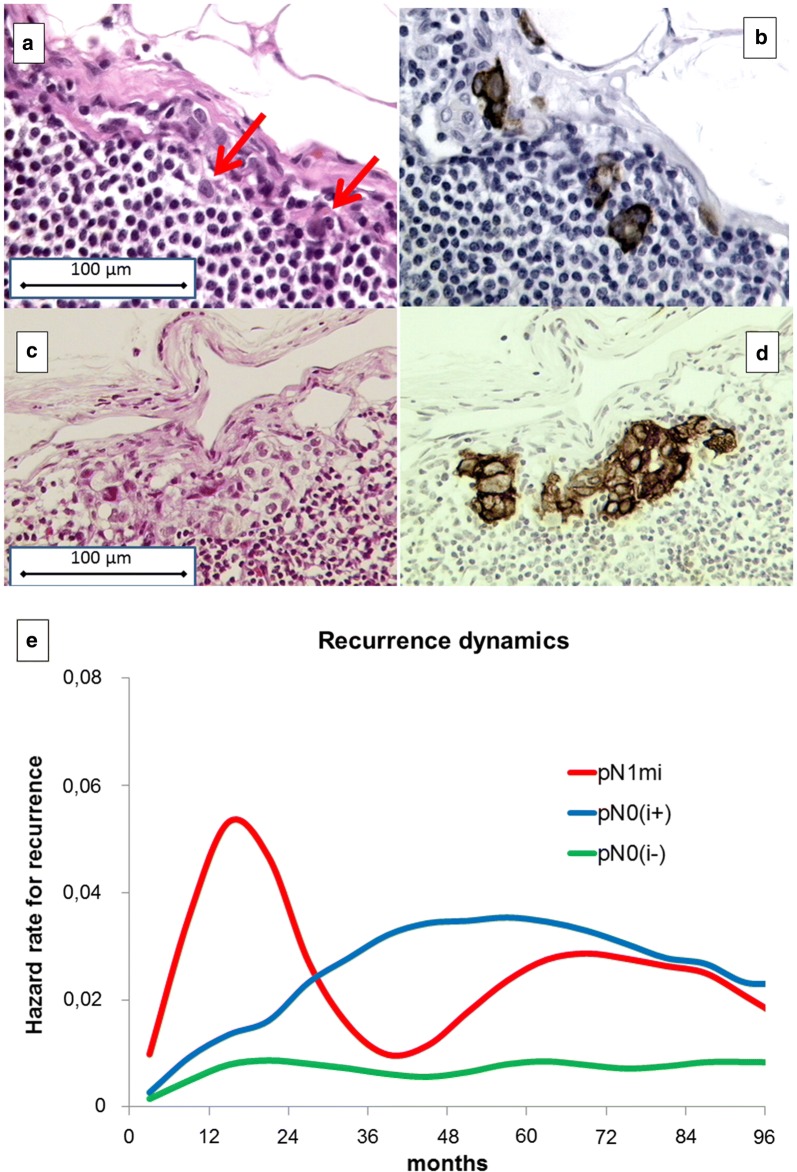
Pathological illustrations of lymph node occult metastases and the different dynamics of disease recurrence in patients with pN0(i−), pN0(i+) and pN1mi early breast cancer. **a**–**d** Examples of lymph node occult metastases from early breast cancer patients analyzed by haematoxylin and eosin (H&E) staining and immunohistochemistry (IHC). Consecutive sections stained by H&E (**a**, **c**) and by IHC (**b**, **d**). The location of the single tumour cells is indicated by arrows. Panels a and b, lymph node pN0(i+) (nanometastases); **c**, **d** lymph node pN1mi (micrometastases). Original magnification 20× objective. e, Hazard rate for recurrence pattern for the investigated patients. There were 328 early breast cancer patients lymph nodes pathologically diagnosed as pN0(i−), 24 as pN0(i+) and 25 as pN1mi. The hazard curve of the pN1mi subcategory displays the usual recurrence pattern as previously observed for breast cancer patients diagnosed with pN1 and pN2 diseases, thereby demonstrating an early peak at the second year and a further increase at about 60 months. By contrast, the recurrence risk curve of pN0(i+) patients, after a relatively stable period during the first 2 years, displays a steady increase and then a plateau-like trend. The recurrence risk pattern for pN0(i−) patients remains largely stable with a hazard rate of about 0.01 recurrence/6 months. pN, pathologically diagnosed nodal subcategories; pN0(i−), no detectable tumor deposits; pN0(i+), lymph nodes containing isolated tumor cells (nanometastases); pN1mi, lymph nodes containing tumour foci with the largest diameter ranging between 0.2 and 2 mm (micrometastases)

**Table 1:** First appearance of clinical events observed during the follow-up period

Events	Undetectable metastases [pN_0(i−)_, *n* = 328]	Nanometastases [pN_0(i+)_, *n* = 24]	Micrometastases [pN_1mi_, *n* = 25]
Local recurrence	20 (17)	3 (3)	5 (4)
Contralateral breast primary	10 (6)	1 (0)	1 (1)
Second primaries^a^	19 (15)	3 (3)	2 (2)
Death	46 (41)	4 (4)	3 (2)
Metastases	29 (25)	7 (6)	4 (3)
Total events	124 (104)	18 (16)	15 (12)

The numbers in parentheses represent events occurred within the median follow-up time of 8 years

pN, pathologically diagnosed nodal subcategories; pN0(i−), undetectable tumor deposits; pN0(i+), lymph nodes containing isolated tumor cells (nanometastases); pN1mi, lymph nodes containing tumour foci with the largest diameter ranging between 0.2 mm and 2 mm (micrometastases)

^a^Other tumours, unrelated to “early breast cancer”, which occured after its diagnosis

In spite of the limited number of events and patients at risk, the hazard rate curves for recurrence (Fig. 1e) show that, in comparison to the recurrence risk of pN0(i−) patients, that of pN0(i+) patients was relatively similar during the first 2 years but displayed a definite level increase afterwards, while that of pN1mi patients demonstrated an early peak followed by a second peak between at about 60 months postoperatively.

The differences in recurrence dynamics, as demonstrated with hazard rate curves, observed between the different subcategories of pN0 early breast cancer patients in this study can be interpretable using the model context of tumour dormancy in specific micrometastatic phases and the occurrence of surgery-related events which have accelerated the metastatic process [4]. The main concept underlying this model is that metastatic foci undergo dormant states, such as single cells in the G_0_ phase of their mitotic cycle, nests containing non-dividing cells, and micrometastases lacking angiogenesis, therefore they are unable to grow to larger than the size of 1–2 mm. Sequential transitions between microscopic dormant states eventually may result in the progressive appearance of metastases when they exceed the clinical detection threshold level. Furthermore, the surgical removal of the primary tumour, the presence of which constrains the development of its microscopic metastases [4], may unlock subclinical tumour dormant foci, thus inducing a sudden acceleration of the metastatic process which manifests during the follow-up, but at different time intervals based on the number of macroscopically non-visible proportions of single dormant cells and avascular micrometastases. These may have resulted in the different recurrence peaks observed between the pN0(i−), pN0(i+) and pN1mi subgroups; paralleling to the number and sequence of metastatic dormancy states. In particular, the early metastasis risk is associated to the sudden switch of angiogenesis in micrometastases, which may be related to surgical manoeuvre, whereas smaller nanometastases need more time to attain the clinical level for visible metastases and thereby display a longer period of dormancy [4]. Therefore, consistently with the lymph node staging, the behavior of hazard rate curves for different subcategories of pN0 early breast cancer patients shown in Fig. 1e suggests the possibility that subclinical metastases in pN0(i+) patients may be mostly comprised of single G_0_ cells or nests containing a few non-dividing cells, whereas in the pN1mi subcategory avascular microscopic foci mostly prevail.

The different dynamics of disease recurrence in patients with pN0(i−), pN0(i+) and pN1mi breast cancer (Fig. 1e) support the notion that the axillary nodal tumour burden, which is a recognized quantitative marker of the recurrence risk at macroscopic level, is also a prognostic factor at microscopic level. This extension of the prognostic role of nodal involvement is revealed by the association of the pathologic findings with the corresponding recurrence hazard rate patterns. This finding suggests that nodal nanometastases can indicate the presence of tumour foci in early dormant states, whereas nodal micrometastases point to dormancy conditions being much nearer to a metastatic-probable clinical manifestation threshold that is reached soon after being surgically stimulated, as has been found to occur in breast cancer patients for whom axillary nodes are macroscopically invaded [4]. Results of the present analyses suggest a correlation between axillary tumour burden and metastatic conditions, starting at microscopic level, thus supporting the concept that axillary lymph nodes may be viewed as accessible sites to assess the biological status of subclinical metastatic deposits in the host. However, such a hypothesis deserves further validation.

## Data Availability

The dataset analyzed during the current study is available from the corresponding author on reasonable request.
